# Delignification of Low-Energy Mechanical Pulp (Asplund Fibers) in a Deep Eutectic Solvent System of Choline Chloride and Lactic Acid

**DOI:** 10.3389/fchem.2021.688291

**Published:** 2021-06-09

**Authors:** Alan D. Pérez, Juha Fiskari, Boelo Schuur

**Affiliations:** ^1^ Sustainable Process Technology Group, Faculty of Science and Technology, University of Twente, Enschede, Netherlands; ^2^ Fibre Science and Communication Network, Mid Sweden University, Sundsvall, Sweden

**Keywords:** deep eutectic solvent, asplund fibers, delignification, lactic acid, choline chloride, pulp quality

## Abstract

Deep eutectic solvents (DESs) are considered as a green and environmentally benign solvent class for various applications, including delignification of biomass. One of the major challenges in the delignification of biomass by DES is attributed to the limitations in mass transfer. By subjecting wood chips to a low-energy mechanical refining, i.e., the Asplund process, the accessible surface area increases greatly, which in turn improves the mass transfer and increases the reaction rate. In this research, the DES delignification of Asplund fibers made of Norway spruce was studied as a strategy to produce papermaking fibers under mild conditions. A DES consisting of lactic acid and choline chloride was used due to its proven performance in delignification. Various operational conditions, such as temperature, time, DES-to-wood ratio, and the type of stirring were studied. A novel parameter, *Q*, allowed to evaluate the impact of the operational conditions on the quality of the pulp in terms of delignification degree and fiber length. The results showed that cooking temperature had the most significant effect on the pulp quality. Additionally, it was observed that cooking times between 30 and 45 min result in a pulp yield of about 50%, while fibers have a lignin content of about 14% and a fiber length of 0.6 mm. These results demonstrate that it is possible to obtain fibers of relatively good quality from DES delignification using Asplund fibers as the starting material.

## Introduction

Wood delignification is conventionally carried out through chemical pulping (CP), where cooking of wood with a chemical solution is performed at high pressure and temperature (Bajpai, 2016; Li et al., 2019). During CP, the chemical structure of lignin is breaking down and solubilizes into the liquid phase. The bonds that link the lignin with the cellulose are broken, allowing also the separation of the cellulose fibers from each other. Through CP processes, a yield between 40–55% (mass of pulp/mass of original fiber source) is achieved, whereas the produced pulp of a good enough quality (given by the delignified high fiber lengths) can be obtained to make office paper and other high-quality papers (Cheremisinoff and Rosenfeld, 2010; Mleziva and Wang, 2012; Bajpai, 2016). The main CP processes used at industrial scale are Kraft, sulfite, neutral sulfite semi-chemical (NSSC), and soda (Cheremisinoff and Rosenfeld, 2010). The most applied technology in the pulp and paper industry is the kraft process (Cheremisinoff and Rosenfeld, 2010; Mleziva and Wang, 2012; Bajpai, 2016). Although highly integrated and allowing for highly efficient recovery of heat by burning the black liquor in the recovery boiler, it is a drawback of the kraft process that most of the lignin is not used for a higher value than its caloric value (*i.e.,* for heat generation). The scientific literature on valorization of chemical constituents of wood is abundant, and both valorization routes for sugar-derived chemicals (Van Putten et al., 2013; Motagamwala et al., 2018) and lignin-derived chemicals (Zakzeski et al., 2010; Li et al., 2015) have been reported. Many of these studies have focused on chemical biorefineries without consideration of the fiber quality, simply because sugars are valorized alternatively, e.g., to make new biopolymers such as polyethylene furanoate (De Jong et al., 2012). More recently, alternative processes have gained attention, aiming to recover lignin as a byproduct, but also to obtain the cellulose fibers.

Organosolv lignin extraction was proposed as an alternative for delignification at mild conditions (Bajpai, 2018; Zijlstra et al., 2020). Usually, the organosolv process is defined as an organic acid-based, alcohol/water-based, and mixed pulping process, which intends to reduce the environmental impact and improve the economy compared to kraft and sulfite processes (Bajpai, 2018). Organosolv is still a pulping process under development based on organic solvents (Bajpai, 2018). Recent studies have been addressing modifications of the organosolv process, such as the organoCat process. It is a biphasic (aqueous and organic) process in which the lignin extraction to the organic phase (containing a homogeneous organic acid catalyst) is carried out simultaneous with selective hemicellulose depolymerization and dissolution in the aqueous phase (Grande et al., 2015). Also processes specifically aiming at dissolution of cellulose fibers have reported (Kilpeläinen et al., 2007; Hauru et al., 2012; Deb et al., 2016), as well as cellulose modification without solubilization (Pena et al., 2019).

Wood delignification through deep eutectic solvents (DESs) has arisen as a potential and green alternative for the pulping process where lignin and hemicellulose (and their derived low molecular weight polymers) are obtained as products in addition to cellulose fibers (Majová et al., 2017; Satlewal et al., 2018; Smink et al., 2019; Fiskari et al., 2020; Jiang et al., 2020). DESs are mixtures of at least two components, one of which acts as a hydrogen-bonding acceptor (HBA), and one is a hydrogen-bonding donor (HBD) (Hansen et al., 2021). The melting point of a DES is lower than either of the individual components due to their strong hydrogen-bonding interaction (Lynam et al., 2017; Schuur et al., 2019). In current studies, several DESs have been tested on the delignification of lignocellulosic materials (Lynam et al., 2017; Majová et al., 2017; Jablonsky et al., 2018; Satlewal et al., 2018; Li et al., 2019; Smink et al., 2019; Fiskari et al., 2020; Jiang et al., 2020; Wang et al., 2021), as well as DES solutions (Soares et al., 2021). Lactic acid and choline chloride (LA:ChCl) DES has shown to be a potential DES for biomass delignification (Jablonský et al., 2015; Alvarez-Vasco et al., 2016; Kumar et al., 2016; Zhang et al., 2016; Liu et al., 2019; Oh et al., 2020) including wood chips (Smink et al., 2019) and Asplund fibers (Fiskari et al., 2020). However, it appears necessary to gain a better understanding about the influence of operational conditions on the quality of the achieved pulp.

Next to the quality of the pulp, also the stability of the DES is of key importance. Recently, it was shown that the DESs comprised of carboxylic acids with ChCl can be unstable due to the esterification of the OH group of the ChCl with the carboxylic acid (Rodriguez Rodriguez et al., 2019). However, when water is present DES systems, degradation by esterification is minimized due to the hydrolysis of the ester being favored by the presence of water (Smink et al., 2020). Indeed, Haz et al. showed that the lactic acid – ChCl DES is stable under pulping conditions up to 190°C (Haz et al., 2016).

During the CP process (kraft, sulfite, organosolv, DES), the chemicals from the bulk liquor must be transported to the active sites within the internal structure of wood (Bogren et al., 2007). Then, the entrapped solution constituted of active chemicals reacts with lignin (Lauwaert et al., 2019) and carbohydrates of the solid matrix of wood (Simão et al., 2008). Thereafter, the degraded and dissolved components of wood must be transported to the surface of the wood chip and consequently to the bulk liquor (Bogren et al., 2007). Several mass transport phenomena are involved in the delignification during chemical pulping by limiting the reaction rates and prolonging the cooking process to a duration of several hours.

Mechanical pulping (MP) is an alternative technology for pulp production that releases the fibers from the wood chips. Among the MP processes, thermomechanical pulping (TMP) is widely used for the manufacture of many paper grades, such as newsprint and lightweight coated (LWC). In the TMP process, after a short preheating in steam, the wood chips are refined in a pressurized system. The first systems to produce MP from chips in a refiner were developed in 1930’s by Arne Asplund in Sweden, but the obtained pulp from the Asplund process was used for fiberboard manufacturing (Höglund, 2009). Asplund fibers require a low specific energy input in refining, typically about 200 kWh/ton. It was not until the 1960’s that the TMP process started to undergo a widespread growth. Since then, it has become the dominant MP process. Unlike CP, TMP provides a high yield (around 90–95 wt%), whereas most of the lignin remains in the produced pulp (Mleziva and Wang, 2012; Bajpai, 2016). A major drawback of TMP is its high energy usage; more than 2.0 MWh/ton for Norway spruce is common (Salmén et al., 1999).

The combination of opening up the structure of the wood by low-energy refining, followed by lignin removal by DES pulping under mild conditions is a novel alternative for the production of delignified fibers for papermaking. However, it is necessary to gain detailed information about operating conditions to develop a sustainable DES pulping process. Based on the experiences with DES-based pulping, knowing that DESs are typically more viscous than traditional aqueous pulping liquors (Andreuccetti et al., 2011; Smink et al., 2019), it may be beneficial for the process if the lignin is made more accessible by a mechanical pretreatment. Therefore, this combination was studied, aiming for a mild DES delignification to obtain fibers with low lignin content. The main objective was to optimize the operating conditions of the DES treatment. The research investigated various operating conditions of DES treatment based on a mixture of lactic acid and choline chloride, which many researchers have studied previously (Majová et al., 2017; Li et al., 2019; Smink et al., 2019; Da Costa Lopes et al., 2020; Fiskari et al., 2020). The investigated parameters included cooking time, cooking temperature, liquor-to-wood ratio (L/W), and the type of mixing (mechanical stirring or a rotatory flask). To calculate the L/W in the cooking experiments, we divided the amount of DES (by weight) by the amount of Asplund fibers (as dry weight). The pulp yield was quantified and the pulp samples were characterized for their lignin content, the amount of fines (wood particles with a size smaller than fibers), as well as fiber length and width.

## Materials and Methods

### Materials

Lactic acid (90% aqueous solution) was purchased from VWR Chemicals. Choline chloride (>98%) and sulfuric acid (95–98%) were purchased from Sigma-Aldrich. After the DES preparation the water content on the DES was measured by Karl-Fischer method by Duplo giving a value of 12.90 ± 0.08 wt%. The Asplund fibers were produced from Norway spruce (see *Methods*) The water content of the Asplund fibers was quantified gravimetrically by weighing its mass on a wet basis and on a dry basis (after oven drying) by triplicate. Mondi AG, Austria, supplied a flash dried kraft pulp sample made of Norway spruce and Scotts pine in the approximate ratio of 3:1. The sample had a kappa number of approximately 45 and it served as a reference. Technical ethanol (100%) purchased by Boom B.V.

### Methods

The Asplund fibers were obtained from a low-energy mechanical pulping process. Afterwards, the Asplund fibers were delignified in the DES solution. The produced cellulose fibers were characterized (in terms of the fiber length and width), and the pulp production was quantified (in terms of yield and pulp lignin content). Additionally, several operating conditions of the cooking process such as cooking temperature and time, L/W ratio, and the kind of mixing were investigated. A detailed description of the methodology of the abovementioned processes is presented in the following subsections.

#### Preparation of Asplund Fibers

Asplund fibers were prepared from Norway spruce (*Picea abies*) chips with a pilot scale refiner at Valmet AB in Sundsvall, Sweden. The objective was to produce wood particles with a large specific surface area to allow rapid reactions between lignin and DESs. The mechanical treatment in Asplund fiber production was similar to the industrial process related to thermomechanical pulp (TMP). These conditions ensure the softening of lignin, which facilitates the fiber separation. The difference is that in the Asplund process, the energy input is very low, which in this case it was 130 kWh/ton. The refining temperature was 165°C. Because the wood chips entering the refiner must have a relatively high moisture, the chips were soaked in water (at room temperature) the night before the refining took place. The heating to the desired temperature was achieved by steam, which also ensured a sufficiently high pressure during the refining.

#### Delignification of Asplund Fibers

Aqueous lactic acid (LA) and choline chloride (ChCl) were added to a 1 L flask in a weight ratio of 10:1 (LA:ChCl), where the weight of LA refers to the weight of the 90% aqueous LA solution. Afterwards, the solution within the flask was mixed at room temperature by magnetic stirring. The agitation was set at 360 rpm in a stirring plate (Salmenkipp) for 1 h, where the ChCl is completely dissolved.

Next, 450 g of the DES was added to a round-bottom flask. Then, the flask with the DES was placed in the cooking setup, and the heating was turned on to reach the cooking temperature (which typically takes 30 ± 5 min). In the meantime, the desired amount of Asplund fibers (16 g < w_AF_ < 30 g) was weighted (reported weight is on a dry basis, the moisture content was 26.24 ± 0.9 wt%) to obtain the desired L/W. Once the DES reached the cooking temperature, the weighted Asplund fibers were added into the round-bottom flask, and the mixing was turned on during the cooking time of the experiment.

After the DES-cooking of Asplund fibers, the solid material was separated from the DES-liquor using a sieve of 53 μm. The solid phase was washed over the sieve with 1 L of a 50 wt% aqueous ethanol solution at room temperature. The solid cooked material was then transferred to a bucket, and 4 L of hot (50–60°C) water was added. The solid material and the hot water were mechanically stirred (with an impeller, controlled by an IKA power control) at a specific speed (275 ± 15 rpm) so that the vortex barely breaks the surface near the shaft of the stirring rod for 5 min.

Afterwards, the solid material and the hot water were poured into a sieve of 200 μm, collecting the washing water in a bucket at the bottom of the sieve. The pH of the washing water into the bucket was measured with pH paper. An empty bucket was placed at the bottom of the sieve, and up to 5 L of hot water was poured in the sieve, and the pH of the washing water into the bucket was measured. This procedure was repeated until the difference of pH between the washing water of the buckets was less than one pH-unit (between 6 and 7 pH). Typically, 12–15 L of hot water was used to reach this point. All solid material over the sieve of 200 μm was collected in a dry, clean, and weighted glass flask of 500 ml. This material was labeled as pulp.

All washing liquids were collected in separate buckets, after which the solid particles smaller than 200 μm were sieved using a sieve of 53 μm. The solids over the sieve were collected in a weighted glass flask of 100 ml. This material was labeled as fines.

The pulp and the fines were oven-dried at 105°C for 48 h. After drying, the weights of the flask with the dry pulp and the flask with the dry fines were measured on a scale (AE Adam, ± 0.01 g), to determine the net weights of the pulp and the fines (both on a dry basis).

#### Variations in Cooking Setups and Conditions

In the delignification experiments, two equipment setups and three operating conditions were investigated. Two cooking setups were used to study the impact of differences in mixing methods. One setup was equipped with mechanical stirring (MS), a heating mantle (Electrothermal), a lid with four necks for the round-bottom flask where a condenser, a thermocouple, and a motor coupled to the impeller were connected to the top of the lid of the flask. A PID was used for temperature control and to set the cooking temperature during the experiment. The stirring was set at 360 rpm. The second setup was a rotary evaporator (RE, Heidolph VV2000) which was equipped with a condenser and a water/oil bath. The temperature was set to the cooking temperature and the speed of rotation of the flask at 360 rpm during the cooking experiment.

All cooking experiments were performed at atmospheric pressure. The impact of cooking temperature on the delignification and fiber length of Asplund was evaluated in both setups for 1 h of cooking. A L/W of 27:1 was used in this set the experiments. The cooking temperatures were 100 and 130°C. This set of experiments were performed in duplicate.

The impact of the L/W was studied using four different levels (15:1, 20:1, 23:1, and 27:1) using the RE set up. The cooking temperature was set to 130°C and the cooking time to 1 h. Additionally, the impact of cooking time (15, 30, 45 and 60 min) on the delignification and fiber length was studied for a L/W of (27:1) and a cooking temperature of 130°C.

#### Lignin Content Quantification

The lignin content in pulp samples and Asplund fibers were quantified by measuring the acid insoluble lignin (AIL) content using the Klason method. Since the extractives had not been removed from the Asplund fibers, the achieved values for the lignin content (LC) from the Klason method is the sum of the AIL, extractives, and ashes (in contrary to some other reported LC values in literature where extensive extractives washing was applied before the LC determination (Sluiter et al., 2012). This difference should be acknowledged when interpreting the results. In this method, 300 ± 10 mg of sample was placed in a pressure tube and hydrolyzed with 3 ml of sulphuric acid (72 wt%) at 30°C (using a water bath) for 1 h. The sample was stirred every 10 min with a stirring rod to ensure all solid material was in contact with the acid. Afterwards, 84 ml of milli-Q water was added to the pressure tube, and it was closed. The closed pressure tube was then placed in an autoclave at 121°C for 1 h. After hydrolysis, the pressure tube was cooled down to room temperature. The material in the pressure tube was filtered using filter paper in a Buchner funnel under reduced pressure. The solids retained on the filter paper were rinsed with 100 ml of milli-Q water. Then, the filter paper with the rinsed solids was placed in an aluminum tray and oven-dried at 105°C until a constant weight was reached. The lignin content was the percentage of the dried solids related to the initial weight of the sample. The LC free of extractives for the raw Asplund was measured by Klason method after an exhaustive Soxhlet extraction in acetone during 24 h.

#### Pulp Fiber Analyses

The fiber length and other fiber characteristics of the achieved pulps were determined with a Valmet Fiber Image Analyzer (Valmet FS5) by Mondi Frantschach GmbH (Austria). Every pulp sample was analyzed in triplo. In this work, the length-weighted average fiber length and the fiber width are reported. The fiber length and width of the raw Asplund fibers was measured in a MorFi analyzer by the French Pulp and Paper Research & Technical Center (CTP, France).

#### Definitions

During the delignification of Asplund fibers, lignin is degraded and dissolved into the DES, and thereby facilitating the liberation of fibers. Additionally, some lignin and wood material are reduced to particles smaller than fibers (53 μm < particle size < 200 μm). The lignin content of the fibers includes AIL, extractives, and the ashes amount. To follow the performance of this delignification process, the pulp yield and the percentage of fines were calculated according to:

$$\text{Pulp}\;\text{yield}\left(\%\right)=\frac{\text{weigth}\;\text{of}\;\text{dried}\;\text{pulp}}{\text{Initial}\;\text{weigth}\;\text{of}\;\text{dried}\;\text{Asplund}}\times 100,$$

$$\text{Fines}\left(\%\right)=\frac{\text{weigth}\;\text{of}\;\text{dried}\;\text{fines}}{\text{Initial}\;\text{weigth}\;\text{of}\;\text{dried}\;\text{Asplund}}\times 100.$$

The degree of delignification (*DD*) and the normalized fiber length (*NFL*) were calculated according to:

$$DD\left(-\right)=\frac{LC_{0}-LC}{LC_{0}},$$

$$NFL\left(-\right)=\frac{FL_{\text{pulp}}}{FL_{\text{ref}}}.$$

The initial lignin content (*LC*_0_) is regarding the lignin content measured at the raw Asplund fibers, while the *LC* is the lignin content on the achieved pulp. The maximum *DD = 1* is achieved when hypothetically the *LC* on the pulp is zero (all lignin is removed), while the minimum *DD* is achieved when no lignin is removed (*DD*_min_ = 0).

The fiber length on the pulp (*FL*_pulp_) is normalized to the reference fiber length (*FL*_ref_). The reference fiber length in this work is the fiber length of the raw Asplund fibers. Although this parameter is normalized, their values are not scaled between zero and one. The maximum value of the *NFL* is achieved when the fiber length of the pulp is the same as the fiber length of reference, which gives a value of the unit (*NFL*_max_ = 1). Moreover, the minimum value of the *NFL* is achieved when the fiber length of the pulp is the minimum. The minimum fiber length was defined as 200 µm (0.2 mm). Particles with smaller sizes are defined as fines. Therefore, the minimum normalized fiber length is given as a function of the reference fiber length, which is constant (*NFL*_min_ = 0.2/*FL*_ref_ [mm]).

The factor between the degree of delignification and the normalized fiber length is defined here as a new parameter (*Q*) which works to quantify the quality of the achieved pulp.

Q(−)=DD×NFL,

with this dimensionless parameter, it is possible to have information about the important variables of the achieved pulp and make easier to stablish the quality of the fibers to define posterior use. Mathematically, the maximum value of *Q* is achieved when both the *DD* and *NFL* are maximum, whereas the minimum value of *Q* is achieved when both the *DD* and *NFL* are minimum.

$$Q_{\max}\{\begin{array}{c} DD_{\max}=1\\ NFL_{\max}=1 \end{array}∴Q_{\max}=1,$$

$$Q_{\min}\{\begin{array}{c} DD_{\min}=0\\ NFL_{\min}=0.2/FL_{\text{ref}} \end{array}∴Q_{\min}=0.$$

Values of the parameter *Q* near to *Q*_max_ means the pulp has a high degree of delignification and high fiber length, while values of the parameter *Q* near to *Q*_min_ indicates the pulp has a low degree of delignification and small fiber lengths.

## Results and Discussion

### Asplund Delignification

Several operating conditions were evaluated for the DES delignification of Asplund fibers. The cooking temperature, the cooking time, the L/W, and two different mixing methods in two different setups. The impact of the cooking conditions was quantified in terms of the pulp yield, percentage of fines, lignin content (which includes the AIL, extractives, and ashes) in the pulp, and the fiber length and width. Lignin content of the raw Asplund was 27.49 ± 0.38 wt%, while LC of raw Asplund free of extractives was 24.83 ± 0.31 wt% (the weight loss of Asplund in dry basis after extractives removal was 4.51 wt%).

**FIGURE 1:**
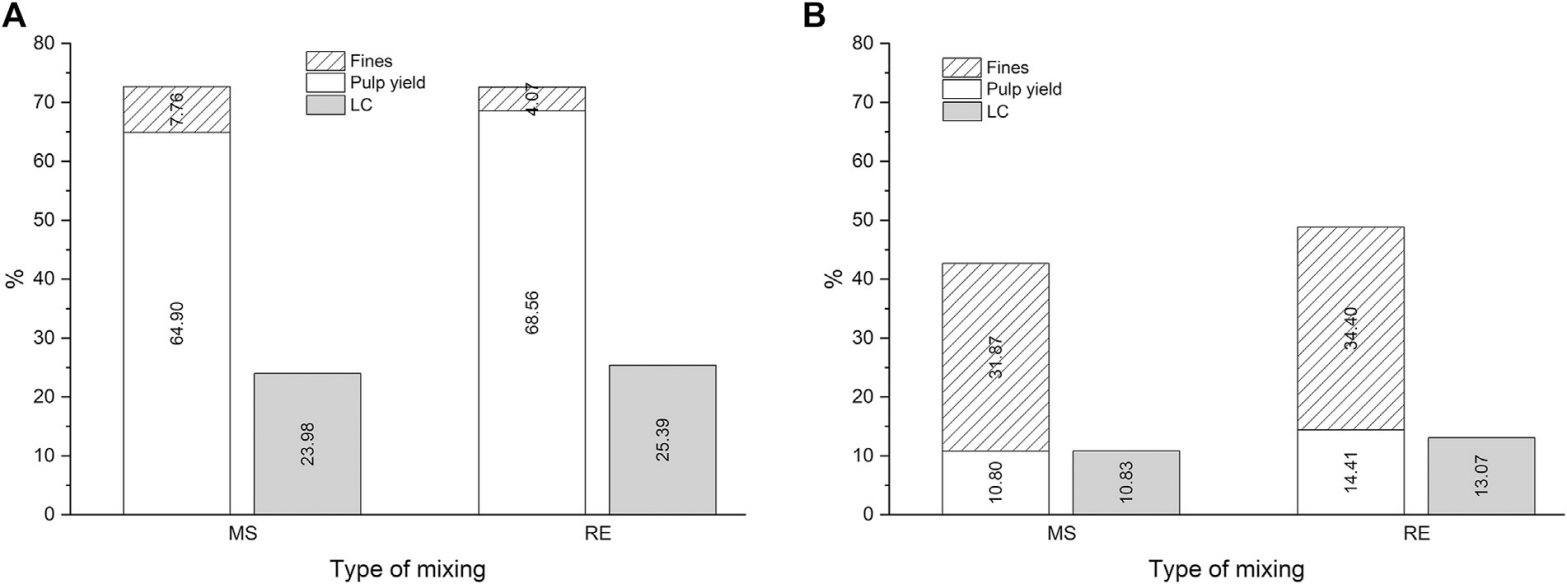
The effect the type of mixing on the pulp yield, fines and lignin content in the DES-cooking of Asplund (1 h of cooking and the L/W at 27:1) by using mechanical stirring (MS) and Rota-evaporator (RE) at 100°C **(A)** and at 130°C **(B)**.

Figure 1 shows the impact of temperature and the type of mixing in the DES-cooking of Asplund fibers at a L/W of 27:1 for 1 h of cooking.

The solid material (pulp + fines) after DES-cooking decreases as the cooking temperature rises. It was reduced from 72.66% at 100°C, to 42.67% at 130°C in the MS set up, and it was reduced from 72.63% at 100°C to 48.81% at 130°C in the RE set up (Figure 1). The LC on pulp was lower at 130°C (10.83 and 13.07% in the MS and RE set up, respectively) than at 100°C (23.98 and 25.39% in the MS and RE set up, respectively) as is shown in Figure 1. Since the LC of the Asplund fibers before the DES-cooking is 27.49 ± 0.38% it is evidenced a low delignification at 100°C, while at 130°C the Asplund fibers are delignified almost to the half of its initial LC, for results achieved from both setups. Despite a notable delignification of the Asplund fibers at 130°C, the amount of the produced pulp is low (10.80 and 14.1% in the MS and RE setups, respectively). According to the results shown in Figure 1, most of the Asplund fibers turned into fines and they were dissolved into the DES (around 85% of the initial amount of the Asplund fibers), when the cooking was performed at 130°C.

The conversion of the main components of wood (lignin, cellulose and hemicellulose) into soluble compounds (low molecular polymers, monomers and decomposition products) into the DES during the DES-cooking process of Asplund fibers is led by three temperature-dependent phenomena:

⁃ Cleavage of β-O-4 ether bonds of the lignin and the subsequent dissolution of the derived polymers on the DES.
⁃ Depolymerization of the cellulose fiber because it is highly exposed surface contact area of Asplund to the DES and the consequent dissolution of the polymers derived from the cellulose into the DES.
⁃ Depolymerization and dissolution of the hemicellulose on the DES.

Both the reaction rates of the involved reactions (during the DES cooking of Asplund fibers) and solubility of solutes (in this case the derived polymers from lignin, cellulose, and hemicellulose) into DES increases as the temperature rises. It is known that significant energy is required for cleavage of the β-O-4 ether bonds in lignin due to the high energy of the C-O bonds in lignin, which is between 218 and 314 kJ mol^−1^ (Jiang et al., 2019; Da Costa Lopes et al., 2020). The DES-cooking at 130°C provides higher energy for the cleavage than the DES-cooking at 100°C. Hence, the conversion of Asplund fibers is higher at 130°C than at 100°C. The conversion of a model compound of the lignin (2-phenoxy-1-phenylethanol) during the DES cooking with LA:ChCl at a molar ratio of 10:1 was studied elsewhere (Da Costa Lopes et al., 2020). They demonstrated that lignin conversion was 51.8% at 120°C, while at 80°C there was no cleavage of the β-O-4 bonds. Although in the work of Da Costa et al., they used a model compound of lignin, and the molar ratio of the LA:ChCl was not the same as in this work, the impact of the temperature on conversion of lignin are in agreement between both investigations. Additionally, the solubility of several monomeric lignin model compounds was proved for several DESs, including LA:ChCl (Soares et al., 2017). These findings support the fact that the cleavage derived lignin molecules may dissolve into the DES.

The reliability of the experiments was measured by the standard deviation (Table 1) of the experiments at different temperatures in both setups, mechanical stirring (MS), and rotary evaporator (RE), which average values are shown in Figure 1.

**TABLE 1:** Yields and standard deviation obtained from the Duplo experiments at 100 and 130°C in both setups during 1 h of cooking at the L/W of 27:1.

	Fines (%)		Pulp yield (%)		LC (%)	
	100°C	130°C	100°C	130°C	100°C	130°C
MS	7.76 ± 2.21	31.87 ± 3.40	64.9 ± 2.63	10.8 ± 2.94	23.98 ± 0.52	10.83 ± 1.04
RE	4.07 ± 1.38	34.40 ± 3.30	68.56 ± 2.07	14.41 ± 2.29	25.39 ± 0.45	13.07 ± 0.72

Standard deviations (SD) for the experiments achieved in the RE setup were lower than those ones achieved in the MS setup, regardless of the cooking temperature. However, the SD between both setups is lower at 100°C than at 130°C. The maximum SD for fines, pulp yield, and LC was 3.40, 2.94, and 1.04%, respectively. The SD of the measurements to quantify pulp and LC among the experiments carried out here is lower than 4%, which shows the reliability of the measurements in this research.

Mixing of the Asplund fibers with the DES in the MS set up is due to the power input of the impeller, while in the RE set up is due to the momentum transfer from the rotary wall of the flask to the DES and Asplund fibers. Despite the speed of mixing in both setups was 360 rpm, the achieved pulp yield was lower by using the MS set up than by using the RE set up, regardless of the cooking temperature. An explanation for the lower pulp yield in the MS is the impeller frequently impacting the Asplund fibers, which makes them weak and break up in their most vulnerable points. Besides, the breaking up of the Asplund fibers due to the mechanical impact of the impeller provided an increase of the surface area of the fibers, which favored the delignification. As a result, the achieved LC on the pulp in the MS set up was lower than the LC achieved in the RE set up, regardless of the applied cooking temperature.

**FIGURE 2:**
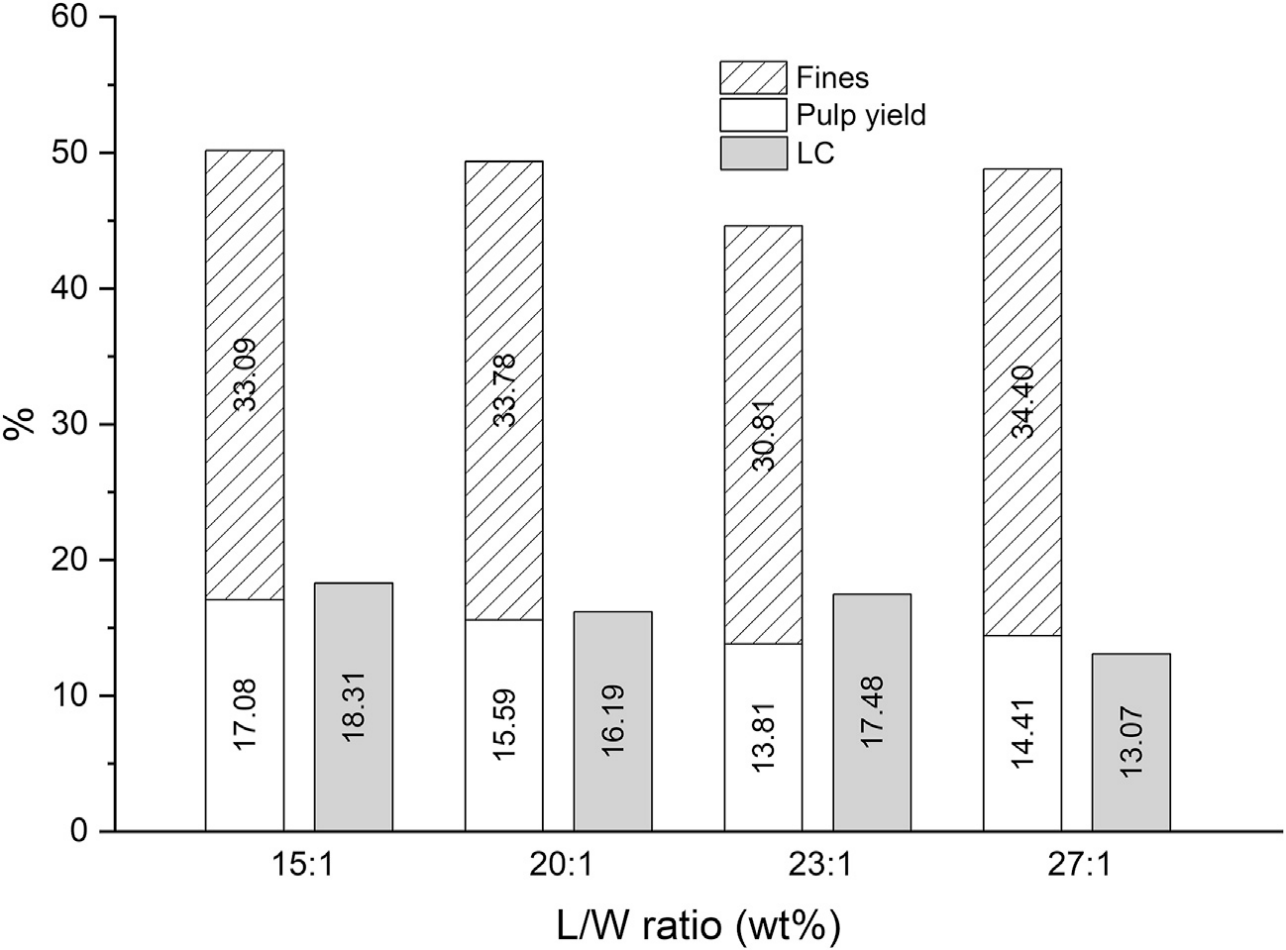
The effect of the L/W on the pulp yield, fines, and the lignin content in the DES-cooking of Asplund fibers (1 h of cooking at 130°C by using the RE setup).

Since the delignification is mild at 100°C and the MS set up has a detrimental impact on the cooked fibers, the effect of the L/W was investigated using the RE setup, and a temperature of 130°C was applied for 1 h of cooking (Figure 2). The amount of fines remained around 33%, the pulp yield around 15% and the LC around 17%. All measured variables remained at the same value within the SD (showed in Table 1), except the LC at the ratio 27:1, which showed slightly lower LC than the mean value achieved at the other tested ratios. Due to the large surface area of Asplund fibers, the mass transfer limitations are much less than with wood chips, where the DES has to penetrate into the wood matrix to delignify it. As a result, the mass transfer limitations also have much less pronounced impact on pulp yield, fines content, and LC. It was observed, however, that the required time for DES to impregnate all of the fluffy Asplund material decreased, as the L/W increased (about 25 min at the lowest ratio, and approximately 10 min at the highest ratio). During 1 h of cooking, there was not a significant impact of the impregnation time on the pulp yield and fines, but a slight effect on the LC was observed at the L/W of 27:1. The cooking time (1 h) is long enough to limit the visibility of the impact of the impregnation time on the effects of process conditions.

**FIGURE 3:**
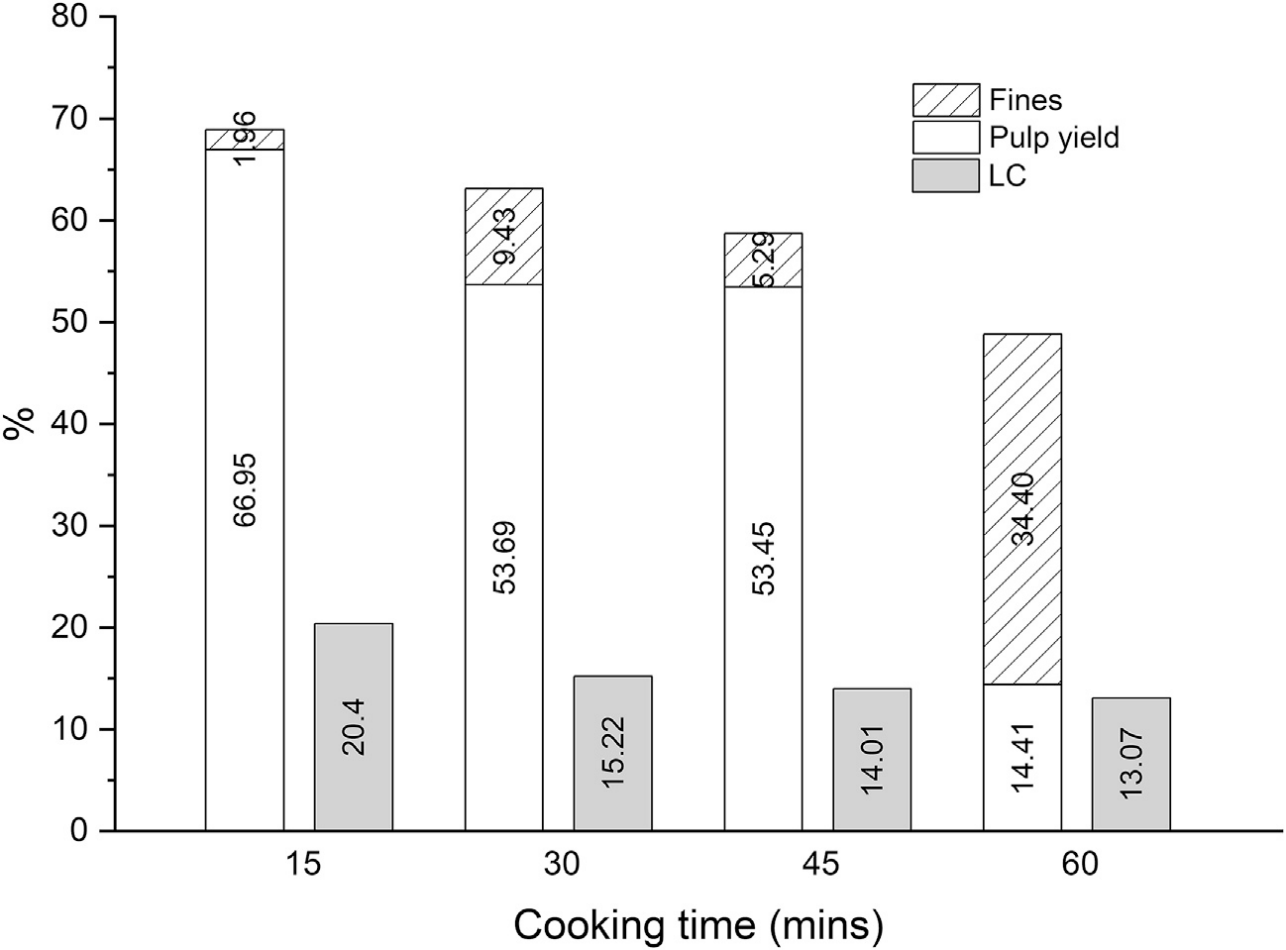
The effect of the cooking time on the pulp yield, fines and lignin content in the DES-cooking of Asplund fibers (the L/W of 27:1at 130°C by using the RE setup).

The impact of the cooking time on the pulp yield, fines, and LC in the DES-cooking of Asplund fibers was investigated at the L/W of 27:1 at 130°C by using the RE set up (Figure 3). At the first 15 min of cooking 68.91% of the initial Asplund remained as solids and 31.09% is dissolved into the DES. From the solid cooked material, 66.95% is pulp with a LC of 20.04% (the Asplund fibers had a LC of 27.49 ± 0.38%). Both at 30 and 45 min of cooking, the obtained solid material is around 60% of the initial mass with a pulp yield and LC around 53 and 14%, respectively. The only difference between 30 and 45 min of cooking is the amount of fines, which is reduced from 9.43% at 30 min to 5.29% at 45 min. After 60 min of cooking the solid materials is slightly lower than 50%, and the pulp yield is decreased to 14.41% with a LC of 13.07%. Most of the solid material at 60 min of cooking consists of fines. Cooking times higher than 45 min do not provide significantly more delignification, but an increased conversion of the pulp into the fines, and the fines into dissolved compounds into the DES.

### Fiber Length and Width

The fiber length and width are two crucial characteristics of cellulose fibers, which may define their potential use, such as the manufacturing of printing papers, packaging boards or tissue. Mondi Frantschach GmbH measured these parameters for the pulps obtained in the experiments using the Valmet Fiber Image Analyzer (Valmet FS5). The fiber area-weighted length and fiber width (by MorFi analyzer) of the raw material were 1.828 mm and 34.28 μm, respectively.

**FIGURE 4:**
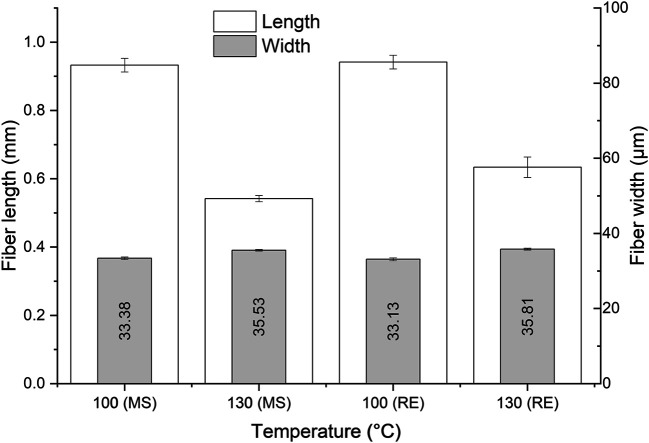
The effect of the temperature and the kind of mixing on the fiber length and width in the DES-cooking of Asplund (1 h of cooking and the L/W at 27:1).

Increasing the cooking temperature had a detrimental impact on the fiber length, as shown in Figure 4. For example using the RE set up, the observed length was 0.942 mm (half of the initial length) at 100°C, and at 130°C it was 0.634 mm only (one third of the initial length). This detrimental impact of the fiber length shows that cellulose depolymerization and the degradation of fibers are important process aspects when using highly solvent-accessible Asplund fibers. On the other hand, the fiber width was hardly affected.

The influence of the kind of mixing (MS or RE) on the fiber length at 100°C was very small, however, at 130°C there was a slight decrease in the fiber length in the MS set up (0.542 ± 0.009 mm) compared to the RE set up (0.634 ± 0.03 mm). It was the mechanical impact that the impeller had on the fibers making them weak in some points, which subsequently were attacked by the DES. Since 100°C is a relatively low temperature for delignification, apparently the fibers were under these conditions resistant enough to limit the fiber shortening effect due to the impact of the impeller.

**FIGURE 5:**
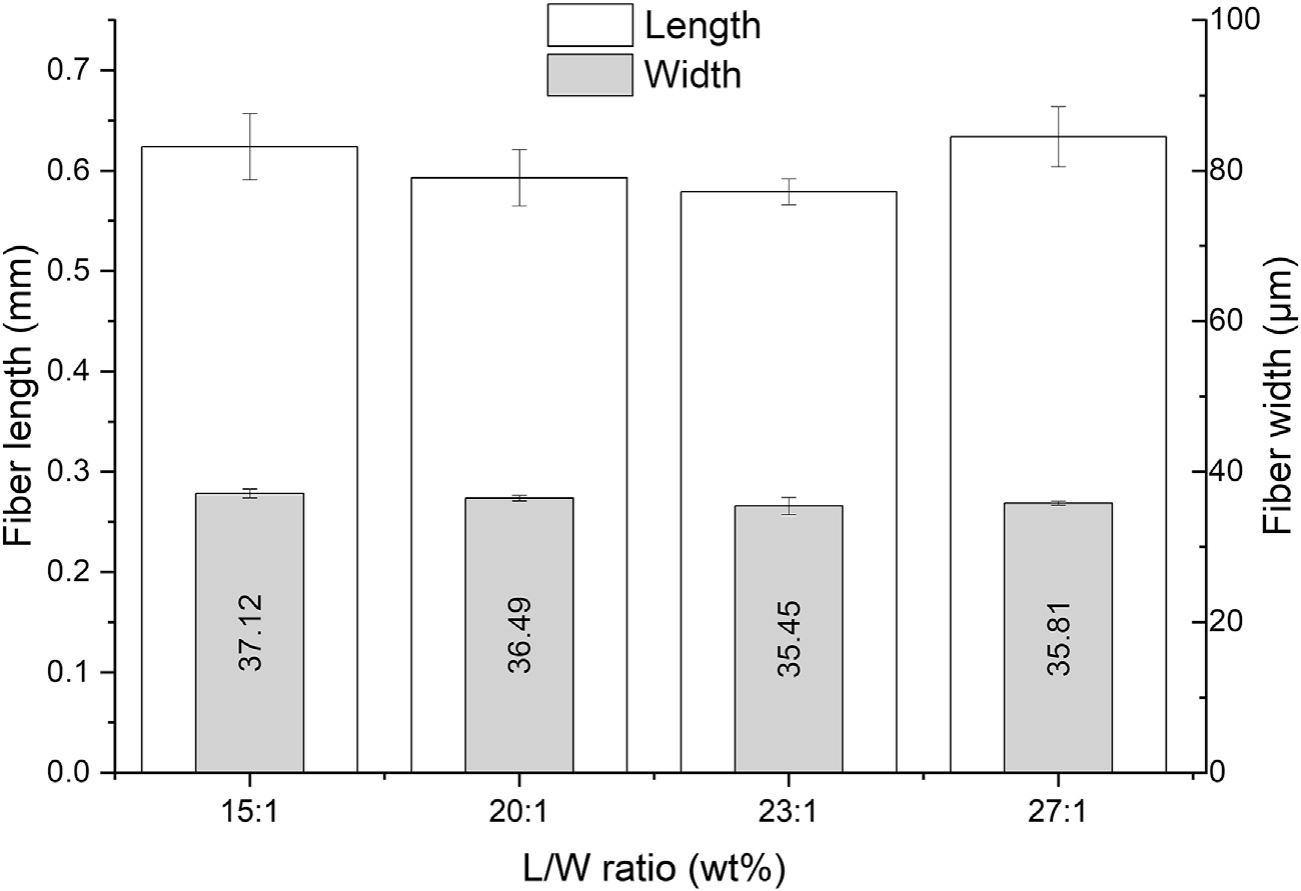
The effect of the L/W on the fiber length and width in the DES-cooking of Asplund (1 h of cooking at 130°C using the RE setup).

Since the L/W mildly affected the pulp yield, also a slight effect on the fiber quality was expected. However, as is shown in Figure 5, the differences were not significant, the fiber length and width remained at a mean value within the SD.

**FIGURE 6:**
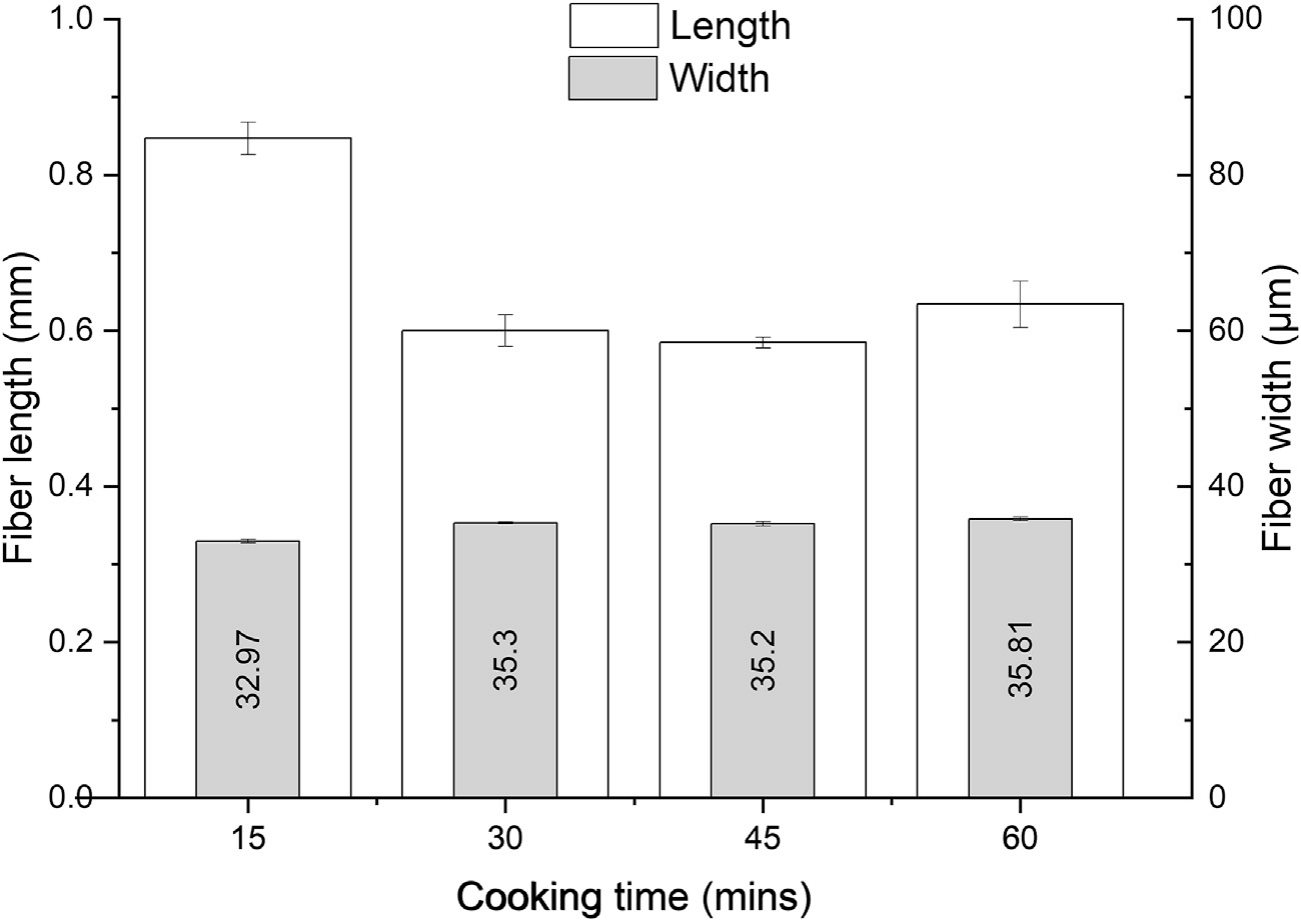
The effect of the cooking time on the fiber length and width in the DES-cooking of Asplund (the L/W of 27:1 at 130°C by using the RE setup).

The most pronounced shortening of the fibers was observed during the first 15 min of cooking (Figure 6). The fiber length was reduced from 1.8 mm (Asplund fibers) to 0.847 mm during this cooking period (a reduction of 53.67%). After 30 min, a plateau of the fiber length (it remained around 0.5 mm) was achieved. Apparently, most weak spots in the fibers were degraded by this time, and the stronger parts of the fibers were resistant enough to have a constant fiber length. Also, the fiber width remained constant over time, which was an indication that mainly the fiber length is a product parameter that is heavily affected by the process conditions.

### Parameterized Results

**FIGURE 7:**
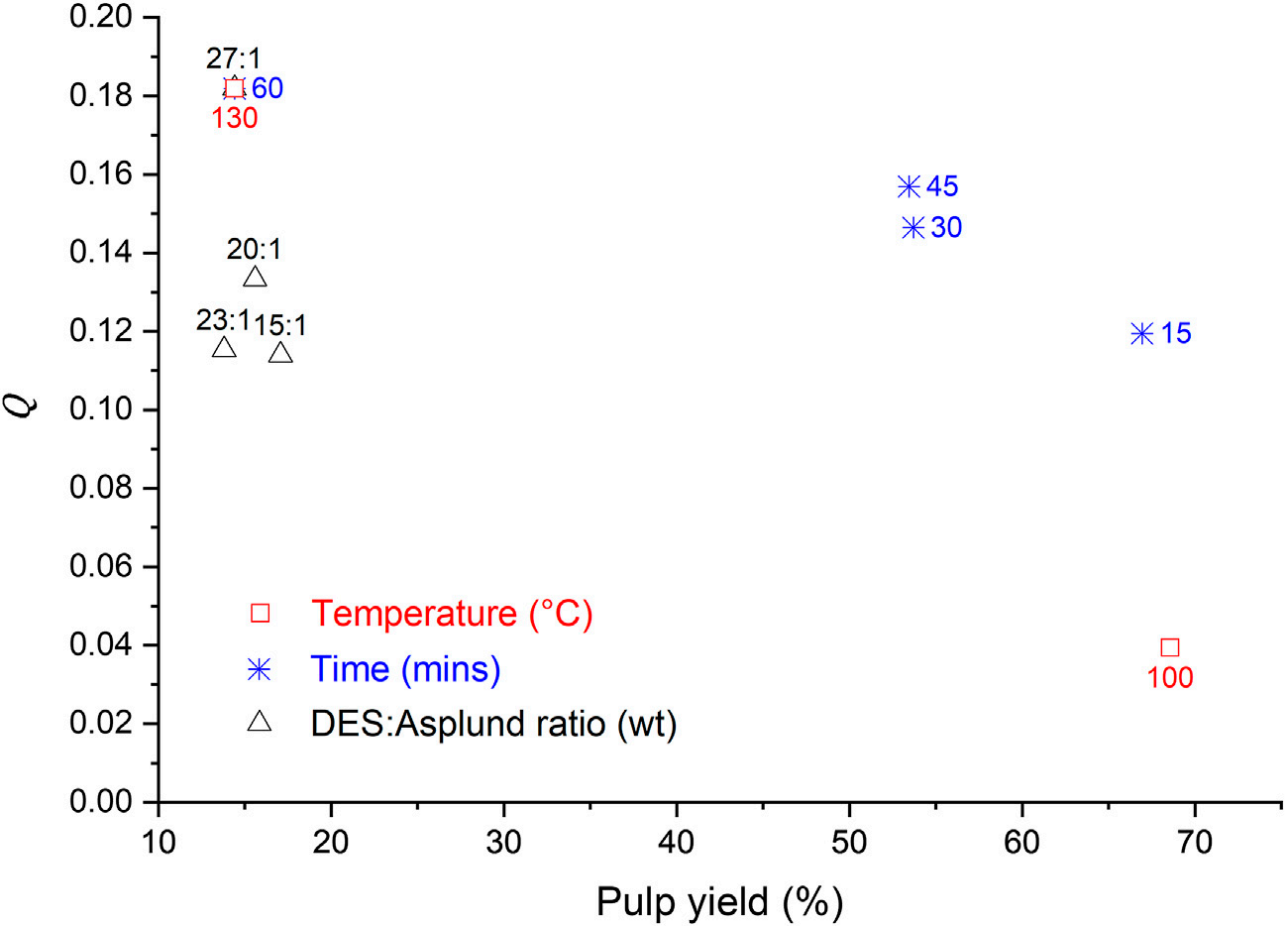
The effect of the cooking temperature, the cooking time, and the L/W on the parameter *Q* in the DES-cooking of Asplund fibers (at the RE setup).

The parametrized results as a function of the pulp yield were analyzed to generate insights in the impact of all tested conditions on the product properties (cooking temperature series, cooking time series, and L/W) using the RE set up. In Figure 7, the parameter *Q* is plotted against the pulp yield for the various series of measurements (time, temperature and L/W).

Both the cooking temperature and the cooking time contributed to significant changes in the *Q* and the pulp yield. However, the cooking temperature was the factor (among all investigated parameters) that had the highest impact on both the *Q* value and the pulp yield. The lowest cooking temperature provided a high pulp yield but a low value of *Q*, whereas the highest cooking temperature provided the highest *Q* value in this investigation but a low pulp yield. On the other hand, cooking times of 30 and 45 min resulted in *Q* values that were nearly the highest ones achieved although the pulp yield was almost 55%. The L/W allowed us to increase the *Q* value without changes in the pulp yield, when the latter was lower than 20%.

For comparison, the *Q* value for unbleached kraft pulp was calculated. The total fiber yield of kraft pulping at a delignification extent of about 90% ranges from 45 to 55%, depending on the wood species and the pulping process (Sixta et al., 2006). The LC of unbleached kraft pulp is usually in the range of 3–5%. However, for a fair comparison the LC of kraft pulp was measured three times using the Klason method, which gave us the value of 12.67 ± 1.98 wt%. This is higher than values reported in the literature, due to the LC in this work have included the extractives and ashes. Extractives are about 3–4 wt% depending of the wood species (Dinwoodie, 2000; Alén, 2015). However, extractives contents in unbleached softwood kraft pulp are typically much lower; for example, Jansson and Nilvebrant (2009) have reported a value of approximately 1%. Therefore, the *Q* value for the unbleached kraft pulp is 0.54, keeping the *NFL* as the maximum. The *Q* value for kraft pulp can be higher if we use the lignin content reported in the literature, as well as another reference for the fiber length, such as that of kraft pulp. However, it is an unequal comparison since the *Q* values for all pulp samples were calculated using LCs measured by the Klason method and the fiber length of the untreated Asplund fibers as the reference. All *Q* values obtained at several cooking conditions in this work were lower than half of the *Q* obtained for the unbleached kraft pulp.

## Conclusion

In this research, a new alternative for chemical pulping was investigated, combining low-energy mechanical pulp (Asplund fibers) and DES (as chemical solution) to produce delignified fibers for papermaking. The impact of the main operating conditions of DES-delignification (cooking temperature, type of mixing, liquor-to-wood ratio, and cooking time) on the quality of produced fibers was studied. The LA-ChCl system was used as the DES benchmark due to its proven capacity to delignify biomass in previous research. The resulting fibers were characterized by measuring their fiber length and width, as well as quantified pulp yield, amount of fines, and lignin content. The standard deviation of the experiments was lower than 3.41%. In addition, the parameter *Q*, which quantifies the quality of pulp, is introduced.

Due to the adverse impact of the impeller on Asplund fibers when using mechanical stirring during the DES-cooking, the rotary evaporator set up was used to investigate the impact of the cooking time and the liquor-to-wood ratio (L/W) on the fiber quality. With a cooking time of 1 h at 100°C and a liquor-to-wood ratio of 27:1, it was possible to achieve a pulp yield of about 65% and a delignification degree lower than 16%. At 130°C, the achieved pulp yield was lower than 15% and the delignification degree was approximately 63%, respectively. In addition, a major decrease in the fiber length was observed with the increase of the temperature, while the fiber width was hardly affected.

According to the observations, there was no significant effect of the L/W ratio on fiber properties with these experimental conditions. This result suggests that the mass transfer limitation is minimal in the delignification of Asplund fibers. This is not the case when wood chips are used, where the L/W ratio has a significant impact on the performance of chemical pulping. This holds true especially for the mass transfer of dissolved material (consisting mainly of lignin and hemicellulose polymers) from the inner part of a wood chip to the bulk of the liquor.

In spite of the low delignification degree (25.79%) when the cooking time was 15 min only and the pulp yield was 66.95%, the resulting fiber length was only half of its initial value. After this initial drastic reduction of the fiber length, we did not observe any significant further decrease when cooking time was extended, suggesting a relatively rapid damage on the weak spots of the fibers.

The parameter *Q*, is a new parameter that combines two relevant variables in the delignification of wood. These are the delignification degree and the normalized fiber length. Values of *Q* near to *Q*_min_ indicate a low delignification degree and a low fiber length. Correspondingly, values of *Q* near to *Q*_max_ imply a high delignification degree and a high fiber length. According to the values achieved of the parameter *Q*, the operational variables with the most pronounced impact on delignification and fiber length were the cooking temperature and the cooking time, achieving a pulp yield higher than 50% and a lignin content lower than 13%. The findings of this work suggest the use of a cooking temperature close to 130°C and a cooking time between 30 and 45 min. However, by controlling both variables, time and temperature, it is possible to optimize the DES-delignification of Asplund fibers to obtain papermaking fibers of reasonable quality.

The achieved experimental Q values of the DES-delignified pulp samples are much lower than the *Q*_max_, which indicates the quality of the produced pulp is relatively low. The main factor appears to be the drastic initial shortening of the fibers due to their weak spots, when subjected to severe cooking conditions. However, several alternatives may be studied in future investigations to improve the quality of the DES delignified fibers. For instance, the Asplund fiber production to obtain fibers with a higher fiber length and less severe mechanical fiber damage (fewer weak spots) can be studied. Moreover, the reactor configuration as well as the process configuration have a significant impact on fiber quality. An interesting alternative is to use a tubular reactor and a continuous process while combining the reactor with the recovery of the DES to feed fresh DES into the delignification. Alternatively, DES with low concentration of dissolved material from delignification can be used.

## Data Availability

The raw data supporting the conclusion of this article will be made available by the authors, without undue reservation.
